# Partially purified *Strongyloides ratti* antigen improved the diagnostic performance of strongyloidiasis by enzyme-linked immunosorbent assay (ELISA) and immunochromatographic test (ICT)

**DOI:** 10.1128/spectrum.02368-24

**Published:** 2025-02-04

**Authors:** Phattharaphon Wongphutorn, Chanika Worasith, Kulthida Y. Kopolrat, Chatanun Eamudomkarn, Opal Pitaksakulrat, Nuttanan Hongsrichan, Patcharaporn Tippayawat, Anchalee Techasen, Jiraporn Sithithaworn, Teeranat Homsombut, Peter Odermatt, Rahmah Noordin, Paiboon Sithithaworn

**Affiliations:** 1 Biomedical Science Program, Graduate School, Khon Kaen University, Khon Kaen, Thailand; 2 Cholangiocarcinoma Research Institute, Khon Kaen University, Khon Kaen, Thailand; 3 Department of Adult Nursing, Faculty of Nursing, Khon Kaen University, Khon Kaen, Thailand; 4 Faculty of Public Health, Kasetsart University Chalermphrakiat Sakon Nakhon Province Campus, Sakhon Nakhon, Thailand; 5 Department of Parasitology, Faculty of Medicine, Khon Kaen University, Khon Kaen, Thailand; 6 Faculty of Associated Medical Sciences, Khon Kaen University, Khon Kaen, Thailand; 7 National Phenome Centre, Khon Kaen University, Khon Kaen, Thailand; 8 Department of Epidemiology and Public Health, Swiss Tropical and Public Health Institute, Allschwil, University of Basel 30247 , Basel, Switzerland; 9 Department of Parasitology and Medical Entomology, Faculty of Medicine, Universiti Kebangsaan Malaysia, Kuala Lumpur, Malaysia; University of Maryland School of Medicine, Baltimore, Maryland, USA

**Keywords:** *Strongyloides stercoralis*, *S. ratti*, antigen fractions, enzyme-linked immunosorbent assay, immunochromatographic test, rapid point-of-care test

## Abstract

*Strongyloides stercoralis* infection is a neglected tropical disease with a global distribution. Serodiagnosis is a sensitive method, but improving its performance and simplifying into a point-of-care test (POCT) are needed. This study aimed to improve the diagnostic performance of serological tests using partially purified *Strongyloides ratti* antigen in an enzyme-linked immunosorbent assay (ELISA) and an immunochromatographic test (ICT). Crude *S. ratti* antigen was purified by an IgG affinity column to partition the antigen into flow-through, washing fraction (WF), and elution fractions. Optimized ELISA and ICT using crude and antigen fractions were used to analyze sera from three groups of subjects. Group 1 comprised subjects with proven strongyloidiasis, Group 2 were subjects with other parasitic infections, and Group 3 were negative parasitic infections. The diagnostic performance and Kappa agreement of the serological tests were analyzed and compared, using larvae detection as the reference test (fecal examination). The results showed that the WF was the most efficient antigen in terms of sensitivity and specificity, as determined by the ELISA and ICT. Kappa’s agreement between fecal examination and WF-ELISA was moderate (Kappa = 0.52), and WF-ICT was almost perfect (Kappa = 0.94). The WF antigen reduced cross-reactivity to other parasitic infections, that is, *Opisthorchis viverrini*, *Taenia* spp., and hookworms, compared to crude *S. ratti* antigen when assessed by ELISA and ICT. We concluded that the WF of purified *S. ratti* improved the ELISA and ICT diagnostic performance, and the latter assay format could be used as a POCT for screening and controlling strongyloidiasis.

**IMPORTANCE:**

This study aimed to improve the serological diagnosis of strongyloidiasis, a disease caused by infection with the intestinal nematode *Strongyloides stercoralis*, by evaluating the impact of *Strongyloides ratti* antigen purification using an IgG affinity column for detecting parasite-specific IgG in serum via enzyme-linked immunosorbent assay (ELISA) and immunochromatographic test (ICT) formats. Compared to crude *S. ratti* antigen, the washing fraction (WF) of the purified antigen demonstrated significantly improved sensitivity and specificity in both ELISA and ICT, achieving strong diagnostic concordance with the gold-standard fecal examination. Furthermore, the WF antigen fraction exhibited reduced cross-reactivity with coinfections caused by the liver fluke (*Opisthorchis viverrini*), tapeworms (*Taenia* spp.), and hookworms. These findings underscore antigen purification as a promising strategy to enhance the accuracy of strongyloidiasis serodiagnosis.

## INTRODUCTION

*Strongyloides stercoralis* is an intestinal parasitic helminth and is widespread globally, including in Southeast Asia, especially Thailand, Lao People’s Democratic Republic, and Cambodia (1–3). Most strongyloidiasis patients are asymptomatic but may exhibit gastrointestinal or dermatological symptoms or progress to highly fatal hyper- or disseminated infections. Effective control and elimination programs require highly sensitive and specific methods for detecting *S. stercoralis* infection. It is known that parasitological examination methods used in the fecal examination are not sensitive. Consequently, several alternative methods have been developed, including serological and molecular techniques. The latter detect parasite-specific DNA by conventional and real-time polymerase chain reaction in feces and urine (4–6). The enzyme-linked immunosorbent assay (ELISA) is the most widely used serological assay for detecting *Strongyloides*-specific antibodies in serum and urine using several antigen preparations. The use of *Strongyloides ratti* as a heterologous antigen for serodiagnosis of strongyloidiasis is preferable compared to *S. stercoralis* due to the ease and safety of the antigen preparation (7). Other sources of antigen, especially recombinant proteins, are also used as alternative materials to *Strongyloides* native proteins for detecting parasite**-**specific IgG and IgG4 antibodies (8, 9).

The use of *S. ratti* as a heterologous antigen for the serodiagnosis of strongyloidiasis presents several advantages over *S. stercoralis*. Large-scale production of *S. stercoralis* filariform larvae relies on human hosts or experimentally infected dogs or primates. This process is not only logistically complex but also poses significant biohazard risks to laboratory personnel. In contrast, *S. ratti* antigens can be safely and efficiently produced using laboratory rats and mice, offering a more secure and practical alternative. Research into the antigenic composition of *S. ratti* extracts supports its potential as a reliable source of antigens for the immunodiagnosis of human strongyloidiasis (7, 8, 10–13). Concerning diagnostic platforms, other than ELISA, the immunochromatographic test (ICT) is a method of choice in developing a rapid diagnostic test (RDT) for serodiagnosis of strongyloidiasis. ICTs have been used in detecting strongyloidiasis using crude *S. stercoralis* and recombinant antigens (14–20). Previous studies have reported several immunodominant antigenic components when using *S. ratti* as an antigen, and cross**-**reactions with other parasitic infections were observed (7, 13, 20, 21). Indeed, these cross**-**reactions were also reported in IgG**-**ELISA using various antigens prepared from *S. stercoralis*, rNIE, and *S. ratti* (8, 13, 22). These results are not unexpected since multiple parasitic infections are common, and shared antigens among the parasites are conceivable. In the case of northeast Thailand, coinfections of *Opisthorchis viverrini* with *S. stercoralis* were commonly observed (7, 13, 20, 23).

Due to the complexity of *S. ratti*’s crude native antigen, we anticipated that partial antigen purification could improve its diagnostic accuracy in detecting strongyloidiasis. Previously, partial purification of *S. stercoralis* antigen to identify fractions that enhanced the diagnostic accuracy of detecting strongyloidiasis had been reported, but they did not include analysis on *S. ratti* (24).

Our study, therefore, focused on the diagnostic performance of partially purified *S. ratti* antigens in detecting strongyloidiasis. We compared this with crude *S. ratti* antigen, employing both ELISA and ICT platforms *for assessment*.

## MATERIALS AND METHODS

### Study design and clinical samples

This study was a diagnostic performance evaluation using retrospective samples. The serum samples were retrieved from the specimen depository of the Cholangiocarcinoma Research Institute, Khon Kaen University, under the project on the liver fluke control program. The project was started from 20 June 2022 to 29 January 2024. The presence of parasitic infections was assessed by fecal examination using the formalin**-**ethyl acetate concentration technique (FECT) (25), and the agar plate culture technique (APCT) (10, 26).

The study participants were separated into three groups based on the results of the fecal examination (FECT and APCT). Group 1 comprised samples from subjects with proven strongyloidiasis (larvae positive) by fecal examination (to determine diagnostic sensitivity), Group 2 comprised samples from subjects with other parasitic infections (excluding *S. stercoralis* infection), and Group 3 comprised samples from subjects negative for parasite infections, that is, “endemic negatives” (to determine diagnostic specificity). Results are reported following the Standards for Reporting Diagnostic Accuracy guidelines (27).

**Fig 1:**
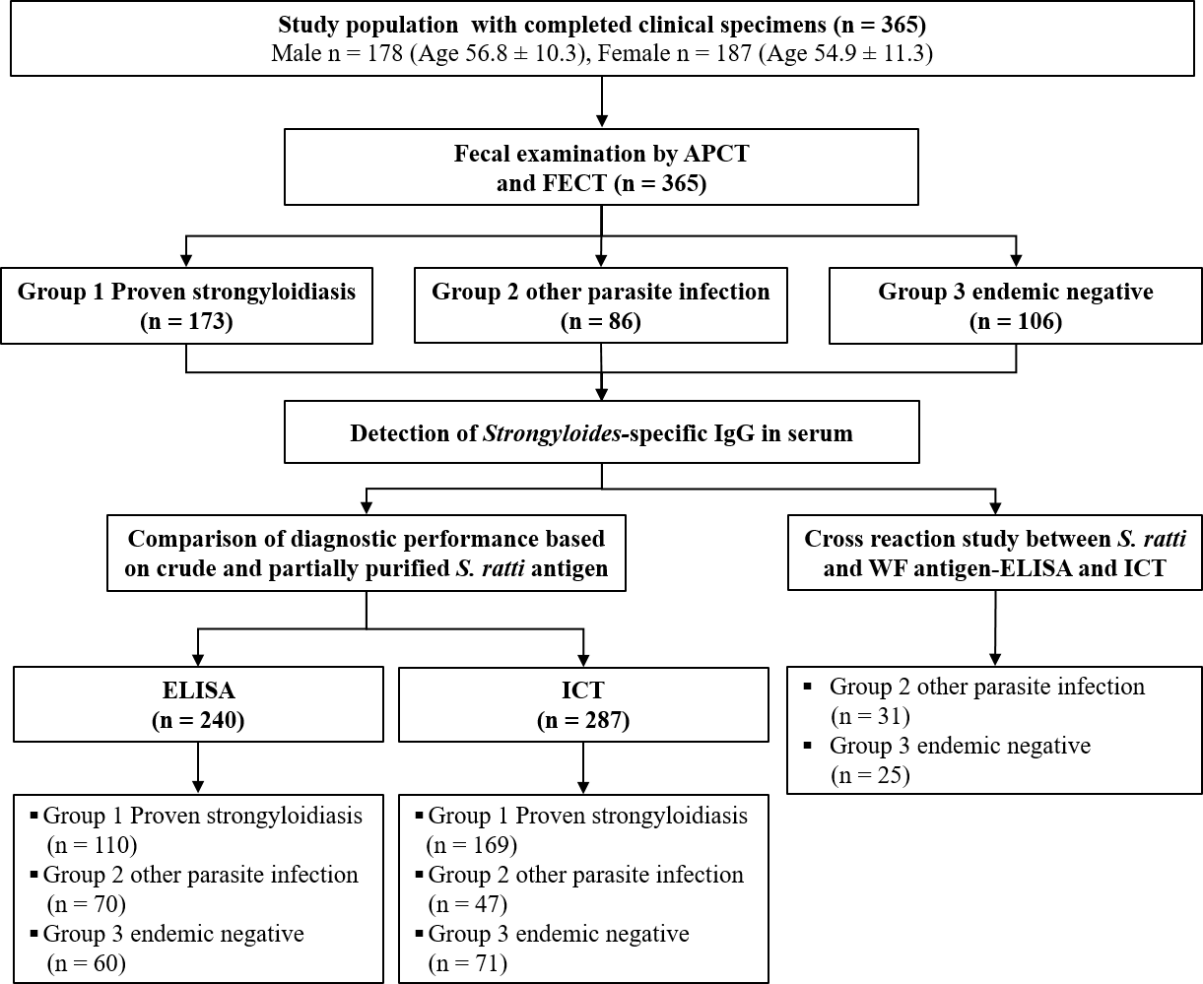
Diagram showing the participants’ groupings, assays performed, and study flow.

In determining the sample size, calculations were carried out using method by Fenn Buderer (28). We assumed that the assays (ELISA and ICT) using the partially purified *S. ratti* antigens have similar sensitivity and specificity as the existing ELISA using crude *S. ratti* antigen (88.1% and 58.8%, respectively) (20). The sample size calculation was based on the proportion of the population expected to be positive for *S. stercoralis* (9%) (29) with the standard score (*Z*-score) set at 1.96, corresponding to a 95% confidence level ±5%. The calculated sample size for diagnostic sensitivity was 96, assuming a sensitivity of 90%. The calculated sample size for diagnostic specificity was 114, assuming a specificity of 60%. Figure 1 shows the participants’ groupings, assays performed, and study flow.

### Preparation of crude *S. ratti* antigen

The life cycle of *S. ratti* was maintained in Wistar rats. The rats were each inoculated subcutaneously with 5,000 *S*. *ratti* third-stage filariform larvae (L3). After 7 days post-infection, fecal samples were examined to confirm the infection, and fecal culture was performed over 20 days by the filter paper culture technique. The supernatant was collected and centrifuged daily, and the pelleted L3 was cleaned under a stereo microscope and then stored at −20°C. Soluble antigen from the L3 was extracted as described previously (30). Briefly, the L3 larvae were washed several times in sterile phosphate-buffered saline (PBS, pH 7.4). Proteinase inhibitor (GE Healthcare, Bio-Sciences Corp., USA) was added to prevent protein degradation. The larvae were disrupted by sonicating for 1 hour at 40 KHz (pulse on 5 s and pulse off 5 s); the homogenate was stored at 4°C overnight and then centrifuged at 15,000 × *g*, 4°C for 30 min. The resulting supernatant (soluble crude antigen) was collected, and the total protein concentration was determined by the Bradford protein assay and then kept at −20°C.

### Partial purification of the crude antigen

The soluble crude *S. ratti* antigen was purified using the Hitrap Protein G High Performace affinity column (Cytiva, Sweden) following the manufacturer’s protocol, with slight modifications. First, the *S. ratti* antigen was diluted (1:1) with the binding buffer (20-mM sodium phosphate buffer, pH 7.0). After mixing, it was filtered through a 0.45-µM syringe filter. The Protein G column was then equilibrated to neutral pH with the binding buffer before sample loading. The antigen solution was then applied to the column at a 1-mL/min flow rate. The flow-through fraction (FF) was collected in 1.5-mL tubes (1-mL/fraction). The column was then washed with five column volumes of the binding buffer at a 1-mL/min flow rate. The unbound or excess protein (washing fraction or WF) was collected in 1.5-mL tubes (1 mL/fraction). Subsequently, bound proteins were eluted using the elution buffer (100-mM glycine-HCl buffer, pH 2.7) and neutralized with 100 µL of 1-M Tris-HCl, pH 9.0. The elution (elution fraction or EF) was collected in 1.5-mL tubes (1 mL/fraction). The total protein concentrations of the fractions were determined by the Bradford protein assay before storage at −20°C.

### Measurements of IgG by ELISA

The unit-based ELISA protocol for IgG detection using the *S. ratti* antigen described previously was used with slight modifications (7, 13). A 96-well plate was coated with 2.5 µg/mL of crude *S. ratti* antigen or partially purified antigen fractions and incubated at 4°C overnight. The plate was washed twice using PBS containing 0.05% Tween-20 (pH 7.2) before blocking with 3% skimmed milk in PBS containing 0.5% Tween-20 at ambient temperature for 2 hours. Serum samples (1:8,000, 100 µL/well) were added and incubated at 37°C for 1 hour. After three times washing of the plates, 100 µL of horseradish peroxidase conjugated to goat anti-human IgG at dilution 1:4,000 (Abcam, USA) was added and incubated at 37°C for 1 hour. After washing three times, O-phenylenediamine dihydrochloride substrate (Sigma-Aldrich, USA) in citrate-phosphate buffer pH 5.0 (100 µL/well) was added. The plate was incubated at room temperature for 1 hour in a humidified dark container. The reaction was stopped by adding 4 N sulfuric acid (50 µL/well), and the optical density (OD) was measured at 492 nm using an ELISA reader (TECAN Sunrise, Austria). The OD was transformed to arbitrary antibody units (per mL or serum) based on a standard curve generated from serial dilutions of pooled positive serum samples.

### RDT based on immunochromatographic technique

**Fig 2:**
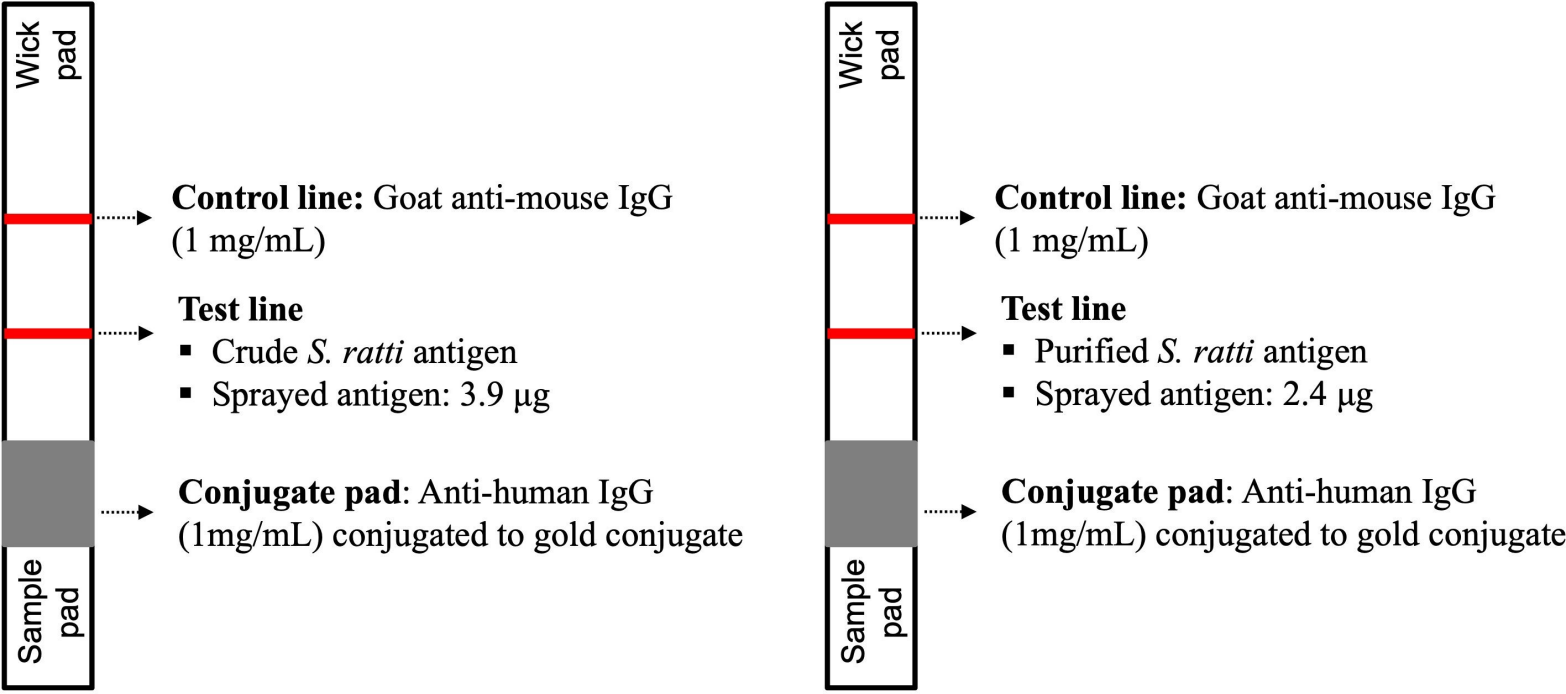
Structure and compositions of ICT cassettes for IgG detection of strongyloidiasis. (**A**) Crude *S. ratti* antigen and (**B**) purified fraction antigen (WF).

The ICT cassette prototype is based on a one-way immunochromatographic technique (Fig. 2). The test cassette consists of pads, filter, and membranes, including nitrocellulose membrane (CN140, Sartorius Stedim Biotech SA, Goettingen, Germany), and the results are seen as test (T) and control (C) lines. Goat anti-mouse IgG at a concentration of 1 mg/mL (Lampire Biological Laboratories, USA) was sprayed at a flow rate of 0.1 µL/mm using XYZ3210 Dispense Platform (BioDot, Irvine, CA, USA) onto the nitrocellulose membrane to form the C line. Crude *S. ratti* antigen (3.5 mg/mL) or partially purified antigen (2.4 mg/mL) was sprayed as the T lines at a flow rate of 0.1 µL/mm. The colloidal gold-conjugated mouse monoclonal anti-human IgG at OD = 10 (Sigma-Aldrich, USA) was sprayed onto a pretreated glass microfiber filter GF33 (Whatman Schleicher & Schuell, Dassel, Germany). The ICT cassettes were produced in collaboration with Kestrel BioSciences Co., Pathumthani, Thailand.

**Fig 3:**
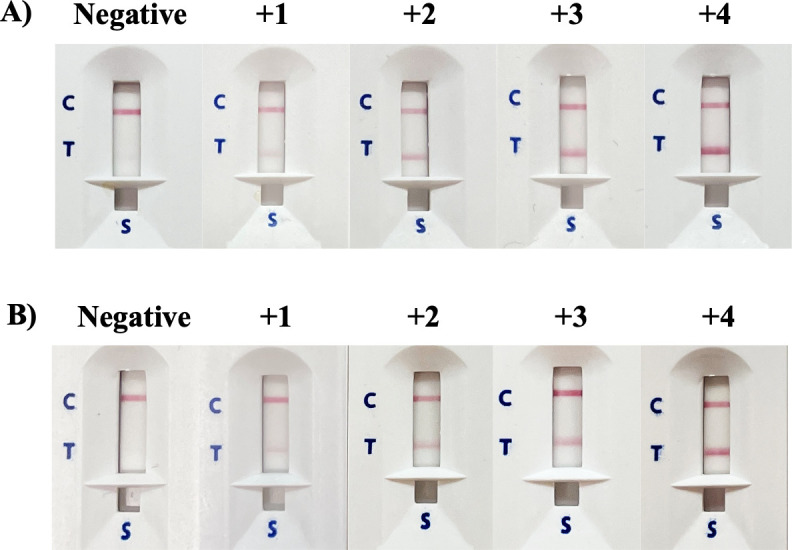
Grading scores of ICT Cassette. (**A**) Crude *S. ratti* antigens and (**B**) WF antigen.

A serum sample (5 µL) was applied to the sample well, followed by adding three drops of running buffer (FL buffer with 0.1% Triton-X 405, pH 8.0) to the same well. *Strongyloides*-specific IgG in the serum sample will covalently bind to the goat anti-human IgG conjugated to colloidal gold before moving upward (via capillary action) to the test and control lines. After 15–20 min, the appearance of red lines at C and T indicates a positive result, and the intensity of the T line is scored visually according to a reference card (grading score +1 to +4). A red line only at C indicates a negative result (Fig. 3).

### Statistical analysis

The cutoff values of *Strongyloides***-**specific IgG by ELISA were determined by the receiver operating characteristic analysis using Medcalc version 11.6.1.0 based on an analysis of 40 proven**-**positive and 40 proven**-**negative serum samples for *S. stercoralis* infection. The area under the curve (AUC) indicates how well a parameter can differentiate between two diagnostic groups, and the cutoff was calculated to achieve the highest sensitivity and specificity (7, 22, 31). The cutoff values in terms of antibody unit/mL of serum for IgG ELISA based on crude *S. ratti* were 144.8, 203.7 for FF antigen, and 190.0 for WF antigen.

Diagnostic sensitivity and specificity of *Strongyloides***-**specific IgG in serum by ELISA and ICT using crude *S. ratti* and partially purified antigens were determined by comparing with the reference test (FECT and APCT). The sensitivity and specificity of each test were also calculated using Medcalc version 11.6.1.0. The data for sensitivity and specificity were compared using the McNemar’s test. Pairwise comparisons of AUC values were evaluated by DeLong’s test (32).

Agreement of diagnostic methods was evaluated using Cohen’s Kappa coefficient (*κ*), for which a value of less than 0.2 indicates a slight agreement, 0.21–0.40 a fair agreement, 0.41–0.60 a moderate agreement, 0.61–0.80 a substantial agreement, and >0.80 an almost perfect agreement (33, 34). The statistical analyses of data obtained in this study were performed using SPSS v.26.0, and the results were considered significant when *P*  <  0.05.

The relationship between positive rates of *S. ratti*-ICT and levels of *Strongyloides*-specific IgG in serum was analyzed using a $\chi^{2}$ test for trend. Data frequency distributions were assessed for normality, and antibody units were normalized using log-transformed data, log (1 + unit) and prior to performing statistical tests and**/**or utilizing nonparametric tests where appropriate. The results were considered significant when *P*  <  0.05.

## RESULTS

### Demographic characteristics and parasitic infections of the study samples

As shown in Table S1, three groups of samples (as described in “Study design and Clinical samples”) for ELISA were Group 1 consisted of 110 samples (67 males, 43 females), Group 2 consisted of 70 samples (38 males, 32 females), and Group 3 consisted of 60 samples (21 males, 39 females). For ICT, Group 1 consisted of 169 samples (98 males, 71 females). Group 2 consisted of 47 samples (19 males, 28 females), and Group 3 consisted of 71 samples (23 males, 48 females). The overlapping samples for analysis by the ELISA and ICT were 56.4% and between different groups were 35%–62%.

### Purification of crude *S. ratti* antigen

#### Purification of *S. ratti* antigen and ELISA optimization

**Fig 4:**
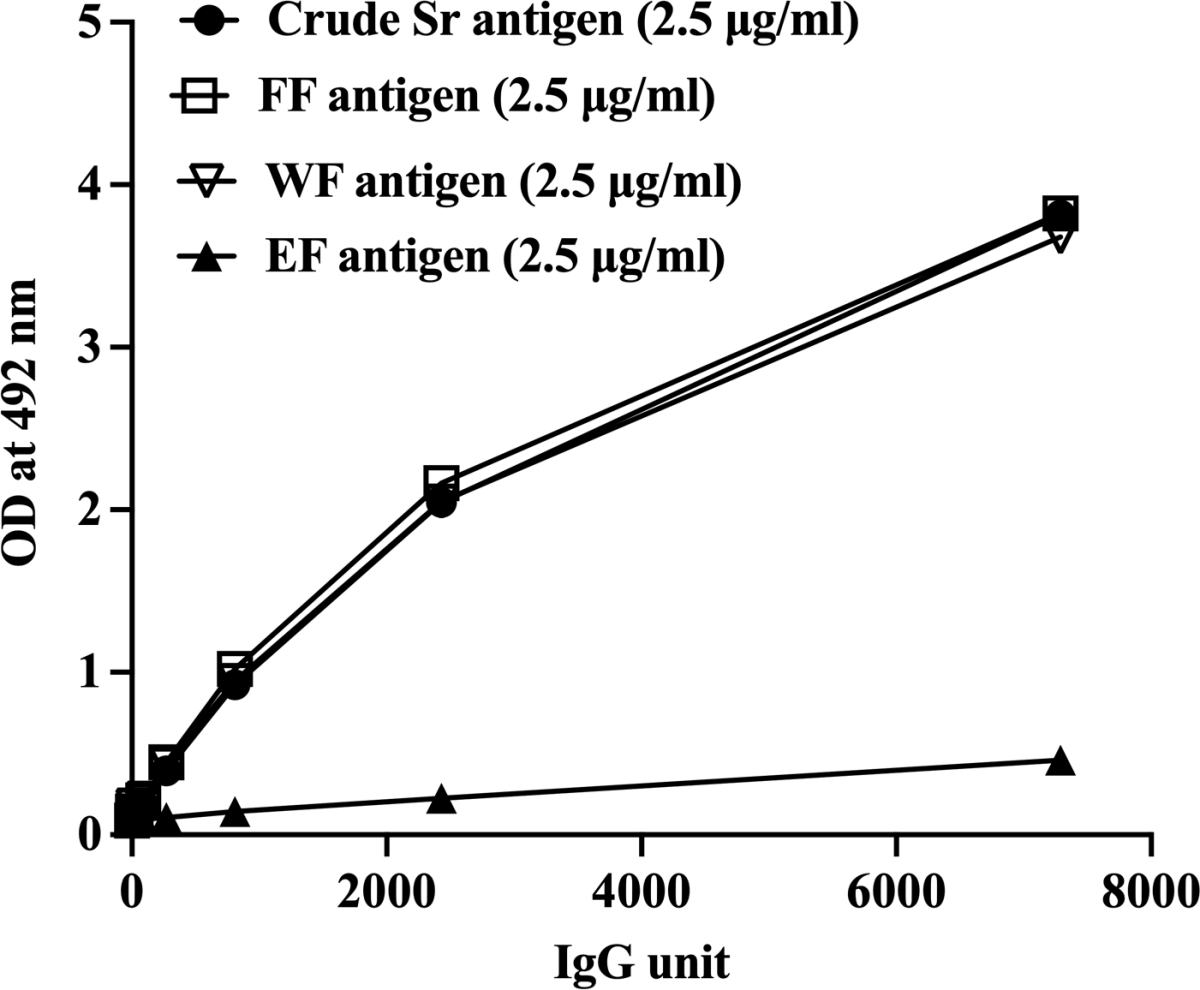
*Strongyloides*-specific IgG levels determined by ELISAs using partially purified (FF, WF, and EF) and crude *S. ratti* antigens and dilutions of positive control sera. Each data point represents a mean of triplicate experiments.

The partially purified *S. ratti* protein comprised three fractions, that is, FF, WF, and EF. The concentration of each antigen fraction was optimized for the measurements of specific IgG in ELISAs and compared with crude *S. ratti*-ELISA using pooled positive and negative *S. stercoralis* sera. Figure 4 shows the results of the optimized ELISAs. It demonstrates that the FF and WF were reactive to *Strongyloides*-positive sera and showed similar profiles to the crude *S. ratti-*ELISA. On the other hand, the EF-ELISA showed low antibody levels; subsequently, only FF and WF of the partially purified antigens were used in the ELISAs.

#### Positive detection rates of strongyloidiasis by ELISAs using crude and partially purified antigens

As shown in Table 1, Group 1 showed 93.6% positive detection by ELISA using crude antigen, 93.6% by FF, and 90.9% by WF (McNemar’s test*, P* > 0.05). Group 2 showed 51.4% positive detection by ELISA using crude antigen, 48.6% by FF, and 45.7% by WF (McNemar’s test*; P* > 0.05). Meanwhile, for Group 3, WF antigen, and not FF antigen, showed significantly lower positive detection compared with crude antigen ELISA (McNemar’s test*; P* < 0.01).

**TABLE 1:** Positive detection rates of *S. stercoralis* infection by crude *S. ratti*-ELISA compared with ELISAs using FF and WF antigen

Group	N	No. positive by crude *S. ratti* ELISA (%)	FF-ELISA		WF-ELISA	
			No. positive (%)	*P* ^*a*^	No. positive (%)	*P* ^*b*^
Group 1 Proven strongyloidiasis	110	103 (93.6)	103 (93.6)	> 0.05	100 (90.9)	> 0.05
Group 2 Other parasite infections	70	36 (51.4)	34 (48.6)	> 0.05	32 (45.7)	> 0.05
Group 3 Endemic negatives	60	26 (43.3)	22 (36.7)	> 0.05	16 (26.7)	< 0.01

^*a*^ Statistically significant difference between crude *S. ratti* and FF-ELISAs by McNemar’s test.

^*b*^ Statistically significant difference between crude *S. ratti* and WF-ELISAs by McNemar’s test.

#### Diagnostic performance of purified and crude *S. ratti* ELISA

Diagnostic sensitivity of IgG**-**ELISA based on the FF and WF antigens was similar, that is, >90% sensitivity (McNemar’s test; *P* > 0.05) (Table 2). The diagnostic specificity of the WF**-**ELISA (63.1%) was significantly increased compared to the ELISAs using crude (52.3%) and FF antigen (56.9%) (McNemar’s test; *P* < 0.001 and *P* < 0.01, respectively). The degrees of Kappa agreement with fecal examination were similar among the ELISAs using FF, WF, and crude *S. ratti* (Table S2). Moreover, ELISAs using crude, FF, and WF antigens showed comparable AUC values (Table S3).

**TABLE 2:** The diagnostic sensitivity, specificity, PPV, and NPV of ELISAs based on crude and purified antigens compared to fecal examination (*n* = 240)

Diagnostic performance test	Fecal examination as reference standard		
	Crude *S. ratti*-ELISA	Purified *S. ratti*	
		FF-ELISA	WF-ELISA
Sensitivity (%) (95% CI)	93.6 (87.3–97.4)	93.6 (8**7**.3–97.4)	90.9 (83.9–95.6)
Specificity (%) (95% CI)	52.3 (43.3–61.1)	56.9 (48.0–65.8)	63.1 (54.2–71.4)
PPV (%) (95% CI)	62.4 (54.6–69.8)	64.8 (56.8–72.2)	67.6 (59.4–75.1)
NPV (%) (95% CI)	90.7 (81.7–96.2)	91.4 (83.0–96.5)	89.1 (80.9–94.7)

### ICT using crude and partially purified *S. ratti* antigens

#### Positive detection rate by crude *S. ratti*-ICT and WF-ICT

In Group 1 of proven *S. stercoralis* infection (*n* **=** 169), the positive rate by ICT using crude and WF antigens was equal at 98.2% (Table 3). However, the false-positive rates when tested with sera from subjects with other parasite infections (Group 2) were 36.2% by crude *S. ratti*-ICT and 10.6% by WF**-**ICT (McNemar’s test, *P* < 0001). Among the endemic negatives of Group 3, the false-positive detection rate was 18.3% by crude *S. ratii*-ICT and 0% by WF-ICT.

**TABLE 3:** Positive detection rates of strongyloidiasis by crude *S. ratti*-ICT and WF-ICT in samples from three groups of participants

Group	N	No. of positive by crude *S. ratti*-ICT (%)	No. of positive by WF- ICT (%)	*P* ^*a*^
Group 1 Proven strongyloidiasis	169	166 (98.2)	166 (98.2)	> 0.05
Group 2 Other parasitic infections	47	17 (36.2)	5 (10.6)	< 0.001
Group 3 Endemic negatives	71	13 (18.3)	0 (0.0)	< 0.001

^*a*^ Statistically significant difference between crude *S. ratti*-ICT and WF-ICT by McNemar’s test.

#### The diagnostic performance of crude *S. ratti*-ICT and WF-ICT

The diagnostic sensitivity of *S. ratti*-ICT using crude and WF antigens was 98.2% (Table 4). Meanwhile, the diagnostic specificity increased from 74.6% with the crude *S. ratti*-ICT to 95.8% with the WF-ICT (McNemar’s test, *P* < 0.001). The diagnostic agreement (Kappa) with reference to fecal examination was almost perfect (Kappa value **=** 0.942, *P* < 0.001) (Table S4). Moreover, there was a significant increase in AUC by the WF-ICT compared to fecal examination (DeLong’s test, *P* < 0.0001) (Table S5).

**TABLE 4:** The diagnostic performance of crude *S*. *ratti*-ICT and WF-ICT antigens compared with fecal examination (APCT and FECT) (*n* = 287)^*a*^

Diagnostic performance test (%)	Fecal examination as reference standard	
	Crude *S. ratti*-ICT	Wf-ICT
Sensitivity (95% CI)	98.2 (94.9–99.6)	98.2 (94.9–99.6)
Specificity (95% CI)	74.6 (65.7–82.1)	95.8 (90.4–98.6)
PPV (95% CI)	84.7 (80.2–88.3)	97.1 (93.4–98.7)
NPV (95% CI)	96.7 **(**90.5–98.9**)**	97.4 (92.5–99.1)
Accuracy (95% CI)	88.5 (84.2–92.0)	97.2 (94.6–98.8)

^*a*^ PPV, positive predictive value; NPV, negative predictive value.

### The correlations between IgG ELISA and ICT based on purified *S. ratti* antigen

**Fig 5:**
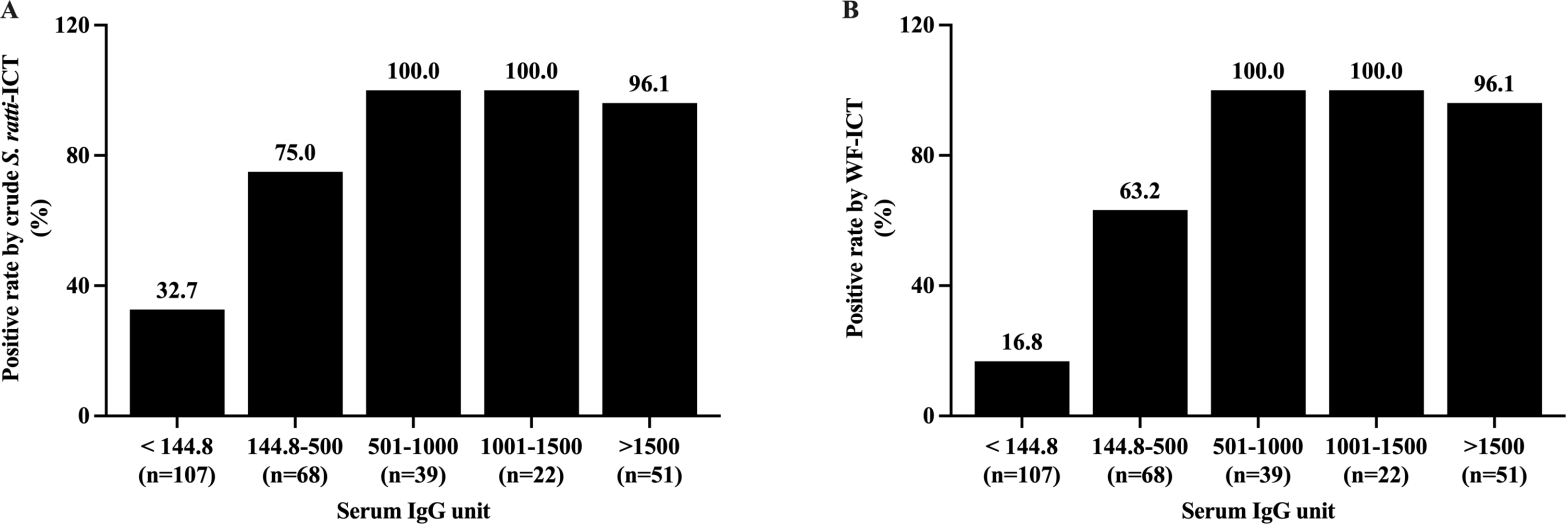
Rates of positive ICT tests for detection of *S. stercoralis* using (**A**) crude *S. ratti* antigen and (**B**) WF antigen, classified by specific IgG**-**ELISA units.

**Fig 6:**
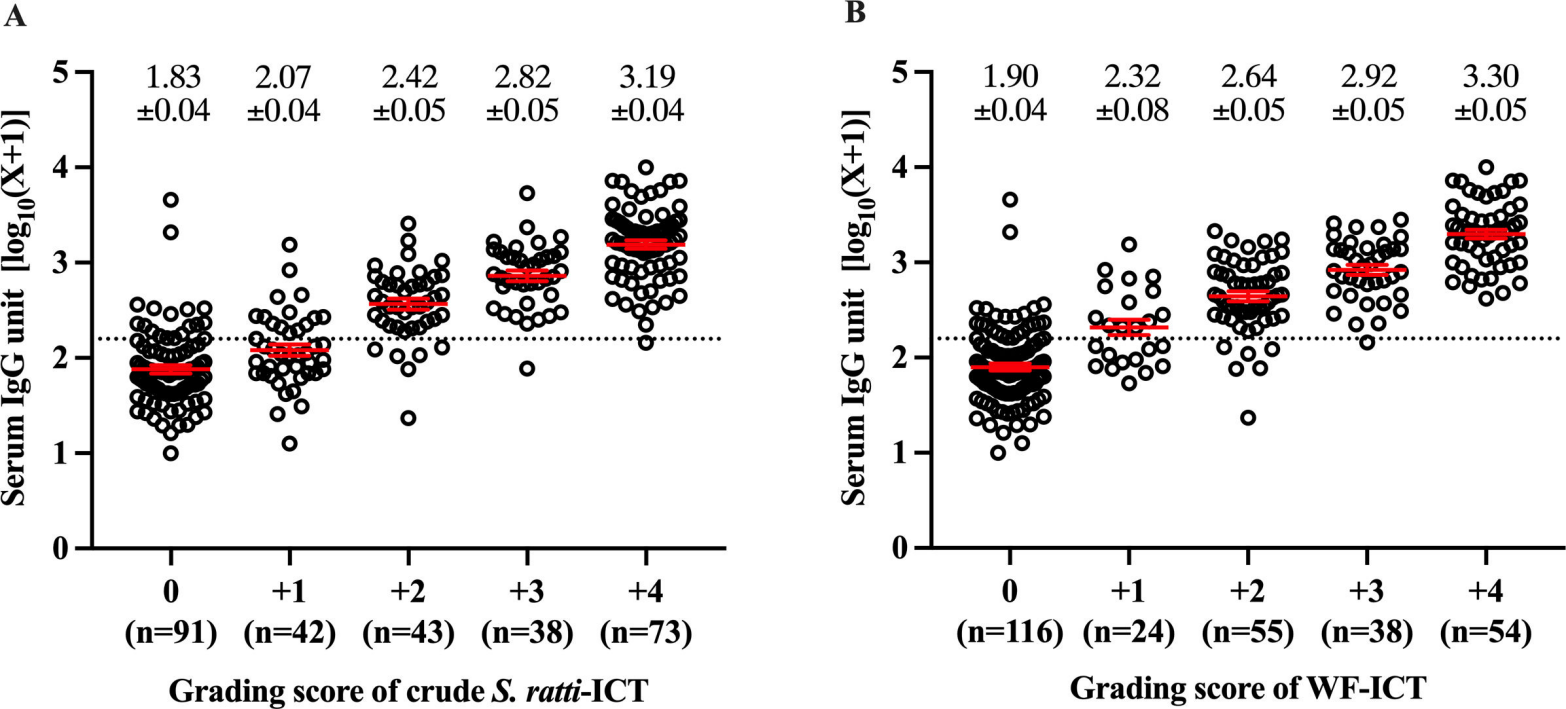
Relationship between levels of serum IgG and ICT grading scores using (**A**) crude *S. ratti* antigen and (**B**) WF antigen. Numbers are the mean ± SE of IgG unit in serum (log-transformed value +1).

The detection rates of ICT based on crude *S. ratti* and WF antigens were positively correlated to IgG antibody units in serum ($\chi^{2}$ test for trend, 83.1—119.4, *P* < 0.0001). Based on the cutoff values of the IgG-ELISA based on crude *S. ratti* (<144.8 units), within the negative IgG-ELISA samples, there were significantly fewer positives by WF-ICT (16.8%, 18**/**107) than by crude *S. ratti*-ICT (32.7%, 35**/**107) ($\chi^{2}$ test **=** 44.5, *P* < 0.001) (Fig. 5). The grading scores of crude *S. ratti*-ICT and WF-ICT were strongly and positively statistically correlated with the levels of *Strongyloides*-specific IgG-ELISA in the samples (Fig. 6, Kruskal–Wallis test, *P* < 0.0001).

### Cross-reaction study between *S. ratti* and WF antigen-ELISA and ICT

As shown in Table 5, the cross-reaction with *O. viverrini* infection by crude-*S. ratti* ELISA was slightly reduced (4.2% less) compared with WF-ELISA. In the case of ICT, there was no cross-reactivity by WF antigen compared with 33.3% by crude-*S. ratti* antigen in *O. viverrini* infection. Based on availability of limited sample sizes (one to three cases), WF-ELISA and WF ICT showed slightly lower cross-reactions for *Taenia* spp., *Trichuris trichiura*, and *Echinostoma* spp. No difference in cross-reaction by crude *S. ratti* or WF-ICT was seen in Minute Intestinal Flukes. Among the endemic negatives (Group 3, *n* **=** 25), the positive rates were reduced from 12% in crude *S. ratti* to 4% in WF-ELISA. In ICT, with reference to the cross-reaction by crude *S. ratti* antigen (20%), no cross-reaction was seen in WF antigen.

**TABLE 5:** The analysis of cross-reactivity based on crude-*S. ratti* and WF of the purified antigen by ELISA and ICT in the samples with other parasitic infections in group 2 (*n* **=** 31) and the endemic negative individuals in group 3 (*n* **=** 25)

Population	N	Positive tests by diagnostic methods (%)			
		Crude-ELISA	WF-ELISA	Crude-ICT	WF-ICT
Group 2					
*O. viverrini*	24	4 (16.7)	3 (12.5)	8 (33.3)	0 (0.0)
*Echinostoma* spp.	1	0 (0.0)	0 (0.0)	1 (100.0)	0 (0.0)
Minute intestinal flukes	2	2 (100.0)	2 (100.0)	2 (100.0)	2 (100.0)
*Taenia* spp.	3	1 (33.3)	0 (0.0)	2 (66.7)	0 (0.0)
*T. trichiura*	1	0 (0.0)	0 (0.0)	1 (100.0)	0 (0.0)
Group 3					
Endemic negative	25	3 (12.0)	1 (4.0)	5 (20.0)	0 (0.0)
Total	56	10 (17.9)	6 (10.7)	19 (33.9)	2 (3.6)

## DISCUSSION

Previous reports showed that several immunodominant antigenic components of *S. ratti* may enhance cross-reactions with other parasitic infections (21). The widely used commercial ELISA kit (Bordier), which uses crude *S. ratti* antigen, was reported to demonstrate up to 11.3% cross-reactivity with other parasitic infections (22). Removing those potentially cross**-**reacting components by purification via protein fractionation may increase the diagnostic specificity of crude *S. ratti* antigen. In the present study, cross**-**reactivity to coendemic parasites using crude *S. ratti* and purified antigen fractions was studied using ELISA and ICT. Previously, our in-house ELISA using crude *S. ratti* antigen showed cross**-**reactions with *O. viverrini* by serum and urine ELISAs (13). The use of defined antigens such as recombinant antigens rNIE, SsIR, and Ss1a by ELISA and ICT also reported some cross**-**reactivity with sera from people with other parasitic infections such as hookworm, trichinellosis, angiostrongyliasis, and capillariasis (8, 9, 14, 18, 19, 35, 36). Thus, improvement of the diagnostic accuracy, particularly the diagnostic specificity of the tests, is needed.

In this study, the crude *S. ratti* antigen purification using a Protein G affinity column yielded three antigen fractions, namely, EF, FF, and WF. After equilibrated with binding buffer, the crude *S. ratti* antigen was loaded into column to collect the FF antigen which consisted of large unbound protein component. The second antigen fraction was the WF antigen which may contain small unbound proteins. Finally, the EF antigen consisting of bound protein and was eluted with glycine-HCl buffer, (pH 2.7) leading to dissociation of protein G and Ig-like protein.

The application of purified WF antigen of *S. ratti* demonstrated high diagnostic sensitivity (98%), comparable to the crude *S. ratti* antigen by both ELISA and ICT. Importantly, the WF-ELISA and WF-ICT assays showed significantly reduced cross-reactivity with other parasitic infections, particularly *O. viverrini* and *Taenia* spp., as well as fewer false positives among endemic negative samples compared to the crude antigen. Notably, the WF-ICT platform exhibited superior diagnostic accuracy (97.2%) compared to WF-ELISA (84.2%), with a diagnostic specificity 20% higher than that of ELISA. These findings were unexpected, as ELISA is generally regarded as more efficient than ICT. While further investigation is warranted, we hypothesize that the improved accuracy of ICT may be due to specific biochemical properties of the purified *S. ratti* antigen. One possibility is that the removal of the IgG-like domain from the crude *S. ratti* antigen through Protein G affinity purification enhances the specificity of the WF antigen. This hypothesis is supported by the fact that Ig-like domains, which are widely distributed across vertebrates, invertebrates, plants, fungi, parasites, bacteria, and viruses, may contribute to cross-reactivity (37). Similar findings, where ICT demonstrated higher sensitivity and specificity than ELISA, have been reported in studies on schistosomiasis (38) and echinococcosis (39).

A previous report of *S. stercoralis* purified by protein size using gel filtration chromatography also improved the diagnostic accuracy of strongyloidiasis by ELISA (24). It was found that F2 (medium size) was the best antigen in the ELISA. The sensitivity and specificity were 95.0% and 96.4%, respectively.

Compared with RDTs based on ICT in previous reports, the crude *S. ratti*-ICT in this study showed 98.2% diagnostic sensitivity, which is higher than ICT using crude *S. stercoralis* (93.3% sensitivity) (17). Furthermore, recombinant antigen-based RDTs were reported to show 91.7% sensitivity for IgG-ICT and varied from 78.3% to 97.0% for IgG4-ICT (14, 16, 19).

Regarding the quantitative aspect of diagnosis, the levels of *Strongyloides*-specific IgG measured by WF-ELISA and WF-ICT scores were quantitatively related, thus suggesting that ICT offers both qualitative and semi**-**quantitative diagnosis of strongyloidiasis. A recently published report on SsRapid IgG4-RDT also showed a significant positive correlation between the RDT scores and *Strongyloides***-**specific IgG and IgG4 levels (10). However, whether the WF-ICT can be used to monitor the outcomes of drug treatment in terms of antibody reduction after treatment remains to be investigated.

We acknowledge several limitations of this study. First, this study used a limited sample size and geographic areas and patient populations. Further investigation is needed to evaluate the use of purified *S. ratti* antigen in diverse settings, including strongyloidiasis-endemic regions such as Africa and Latin America, as well as non-endemic areas in developed countries like Europe and North America. Second, in order to rule out possible cryptic strongyloidiasis in sample Group 2 (other parasites) and Group 3 (endemic negative), repeated stool examination by APCT and FECT and confirmation by molecular detection methods (2, 6) in cases of false-positive tests should have been done to better evaluate the performance of parasite-specific IgG detection by ELISA or ICT. Lastly, we did not perform immunoblotting to elucidate the specific antigenic bands of the fractions, particularly WF; hence the target antigen(s) which are reactive to IgG detection remained unknown. An in-depth protein analysis study is thus warranted.

In conclusion, the antigen purification approach using the Protein G affinity column yielded a promising antigen fraction (WF) that improved the diagnostic specificity and maintained high diagnostic sensitivity by ELISA and ICT. Since *S. ratti* antigen preparation is not hazardous and can be readily produced in laboratory animals, the above approach is feasible for an adequate supply of the WF antigen. The corresponding recombinant protein(s) to the antigenic component(s) can be produced to provide an unlimited antigen supply and perhaps enhance the diagnostic performance.

Pending further studies in other *Strongyloides* endemic areas, using *S. ratti* WF antigen is promising for improving the diagnostic specificity and accuracy of *Strongyloides* serodiagnosis.

## Data Availability

The data that support the findings of this study are available from the corresponding author upon reasonable request.

## SUPPLEMENTAL MATERIAL

Supplemental tables spectrum.02368-24-s0001.docx Tables S1 to S5.

ASM does not own the copyrights to Supplemental Material that may be linked to, or accessed through, an article. The authors have granted ASM a non-exclusive, world-wide license to publish the Supplemental Material files. Please contact the corresponding author directly for reuse.
