# Supplementary material for: Partially purified Strongyloides ratti antigen improved the diagnostic performance of strongyloidiasis by enzyme-linked immunosorbent assay (ELISA) and immunochromatographic test (ICT)
Source: Microbiol Spectr. 2025 Feb 4;13(3):e02368-24. doi: 10.1128/spectrum.02368-24 (PMC11878071; doi:10.1128/spectrum.02368-24)
Supplement: Supplemental tables — Tables S1 to S5. [file spectrum.02368-24-s0001.docx]

**Table S1** The groupings of participants and numbers of serum samples used for diagnostic performance analysis for strongyloidiasis by ELISA and ICT.

| **Population** | **Number of study participants**  **for immunological analysis** | | |
| --- | --- | --- | --- |
|  | **Serum ELISA** | **Serum ICT** | **Overlapping (%)** |
| **Group 1 proven strongyloidiasis**   - No. of participants - Male - Female - Age **(**mean ± SD) | 110  67  43  57.7 ± 9.4 | 169  98  71  57.9 ± 9.7 | 106 **(**62.7**)**  63  43  57.1 ± 9.7 |
| **Group 2 other parasite infection**   - No. of participants - Male - Female - Age **(**mean ± SD) | 70  38  32  56.5 ± 8.6 | 47  19  28  57.2 ± 10.3 | 31 **(**44.2**)**  14  17  56.2 ± 9.8 |
| **Group 3 endemic negative**   - No. of participants - Male - Female - Age **(**mean ± SD) | 60  21  39  52.9 ± 10.4 | 71  23  48  49.3 ± 13.7 | 25 **(**35**.**2**)**  11  14  48.2 ± 10.3 |
| **Total** | **240** | **287** | **162 (56.4)** |

**Table S2** Agreements **(**Kappa**)** of diagnosis of strongyloidiasis between the fecal examination **(**APCT and FECT**)** and IgG ELISAs based on crude and partially purified *S****.*** *ratti* antigens.

| **Methods** | **Fecal examination as reference standard** | | | |
| --- | --- | --- | --- | --- |
|  | **Kappa (*κ*)** | **95% CI (Lower-Upper)** | ***P-*value** | **Level of**  **agreement** |
| Crude *S****.*** *ratti****-***ELISA | 0**.**442 | 0**.**336**-**0**.**543 | < 0**.**001 | moderate |
| FF-ELISA | 0**.**489 | 0**.**381**-**0**.**597 | < 0**.**001 | moderate |
| WF-ELISA | 0**.**526 | 0**.**422**-**0**.**632 | < 0**.**001 | moderate |

**Table S3** The ROC curve analysis of the IgG ELISAs IgG ELISAs based on crude and partially purified *S****.*** *ratti* antigens.

| **Methods** | **Fecal examination as reference standard** | | | |
| --- | --- | --- | --- | --- |
|  | **AUC** | **95% CI**  **(Lower -Upper)** | ***P*-value** | **Pairwise comparison**  **With crude antigen** |
| Crude *S****.*** *ratti****-***ELISA | 0**.**730 | 0**.**669**-**0**.**785 | < 0**.**001 | **-** |
| FF-ELISA | 0**.**753 | 0**.**693**-**0**.**806 | < 0**.**001 | P = 0**.**3030 |
| WF-ELISA | 0**.**770 | 0**.**711**-**0**.**822 | < 0**.**001 | P = 0**.**0557 |

**Table S4** Diagnostic agreement of ICT based on crude *S*. *ratti-*ICT and WF-ICT antigens

compared with fecal examination **(**APCT and FECT**)**.

| **Methods** | **Fecal examination as reference standard** | | | **Level of**  **agreement** |
| --- | --- | --- | --- | --- |
|  | **Kappa (*κ*)** | **95% CI**  **(Lower-Upper)** | ***P* value** |  |
| Crude *S. ratti*-ICT | 0.754 | 0.676-0.829 | < 0.001 | Substantial |
| WF-ICT | 0.942 | 0.901-0.978 | < 0.001 | Almost perfect |

**Table S5** The ROC curve analysis of serum IgG detection by ICT based on crude and washing fraction of purified antigens compared with fecal examination **(**APCT and FECT**).**

| **Antigens** | **Fecal examination as reference standard** | | |
| --- | --- | --- | --- |
|  | **AUC**  **(95% CI)** | ***P* value** | **Pairwise comparison**  **of ROC curves** |
| Crude *S. ratti*-ICT | 0**.**864 (0**.**823-0**.**905**)** | < 0**.**0001 | *P* value < 0**.**0001 |
| WF-ICT | 0**.**970 **(**0**.**949-0**.**991**)** | < 0**.**0001 |  |
